# Downregulation of genes with a function in axon outgrowth and synapse formation in motor neurones of the VEGF^δ/δ ^mouse model of amyotrophic lateral sclerosis

**DOI:** 10.1186/1471-2164-11-203

**Published:** 2010-03-26

**Authors:** Alice Brockington, Paul R Heath, Hazel Holden, Paul Kasher, Florian LP Bender, Filip Claes, Diether Lambrechts, Michael Sendtner, Peter Carmeliet, Pamela J Shaw

**Affiliations:** 1 Academic Neurology Unit, University of Sheffield, E Floor, Medical School, Beech Hill Road, Sheffield S10 2RX, UK; 2 Institute for Clinical Neurobiology, University of Würzburg, D-97080 Würzburg, Germany; 3 Vesalius Research Centre, VIB - KULeuven, Leuven, Belgium

## Abstract

**Background:**

Vascular endothelial growth factor (VEGF) is an endothelial cell mitogen that stimulates vasculogenesis. It has also been shown to act as a neurotrophic factor *in vitro *and *in vivo*. Deletion of the hypoxia response element of the promoter region of the gene encoding VEGF in mice causes a reduction in neural VEGF expression, and results in adult-onset motor neurone degeneration that resembles amyotrophic lateral sclerosis (ALS). Investigating the molecular pathways to neurodegeneration in the VEGF^δ/δ ^mouse model of ALS may improve understanding of the mechanisms of motor neurone death in the human disease.

**Results:**

Microarray analysis was used to determine the transcriptional profile of laser captured spinal motor neurones of transgenic and wild-type littermates at 3 time points of disease. 324 genes were significantly differentially expressed in motor neurones of presymptomatic VEGF^δ/δ ^mice, 382 at disease onset, and 689 at late stage disease. Massive transcriptional downregulation occurred with disease progression, associated with downregulation of genes involved in RNA processing at late stage disease. VEGF^δ/δ ^mice showed reduction in expression, from symptom onset, of the cholesterol synthesis pathway, and genes involved in nervous system development, including axonogenesis, synapse formation, growth factor signalling pathways, cell adhesion and microtubule-based processes. These changes may reflect a reduced capacity of VEGF^δ/δ ^mice for maintenance and remodelling of neuronal processes in the face of demands of neural plasticity. The findings are supported by the demonstration that in primary motor neurone cultures from VEGF^δ/δ ^mice, axon outgrowth is significantly reduced compared to wild-type littermates.

**Conclusions:**

Downregulation of these genes involved in axon outgrowth and synapse formation in adult mice suggests a hitherto unrecognized role of VEGF in the maintenance of neuronal circuitry. Dysregulation of VEGF may lead to neurodegeneration through synaptic regression and dying-back axonopathy.

## Background

Amyotrophic lateral sclerosis (ALS) is a fatal neurodegenerative disorder in which selective loss of motor neurones in the cerebral cortex, brainstem and spinal cord leads to progressive paralysis. In the majority of cases of ALS, the cause of motor neurone degeneration is unknown, although a number of pathogenic processes, including oxidative stress, excitotoxicity, inflammation, and mitochondrial and neurofilament dysfunction, are thought to play important roles. Ten percent of cases of ALS are familial, and in 20% of these, a causative mutation is found in the gene encoding superoxide dismutase I (SOD1), a free radical scavenger. In the SOD1 rodent model of ALS, overexpression of human mutant SOD1 causes adult onset motor neurone degeneration.

Vascular endothelial growth factor (VEGF) is an endothelial cell mitogen that stimulates angiogenesis in response to hypoxia, in the developing embryo and in a number of pathological conditions, such as tumour growth. VEGF transcription is upregulated by binding of hypoxia inducible factor (HIF-1) to a hypoxia response element (HRE) in the 5' promoter region of the gene. In 2001, Oosthuyse *et al *deleted the HRE of the VEGF gene in mice, to generate VEGF^δ/δ ^mice [1], which express reduced levels of Vegf in neural tissue under both baseline and hypoxic conditions. VEGF^δ/δ ^mice show 60% mortality at or around birth, and surviving transgenic mice are slightly smaller than their wild-type littermates. At 5 months of age, they develop a motor neurodegenerative phenotype that resembles ALS, with impairment of motor behaviours and motor tests, such as the treadmill test. Electrophysiological studies show signs of denervation and compensatory reinnervation, muscle histology shows neurogenic atrophy, and peripheral nerves show loss of large myelinated motor axons. In the spinal cord and brainstem, similar numbers of motor neurones are present until 3 months of age, but by 17 months, there is a 30% reduction in motor neurone numbers, with a reactive astrocytosis, and neurofilament inclusions in surviving neurones [1]. The mechanism of neurodegeneration in VEGF^δ/δ ^mice is unknown. Chronic hypoxia has been proposed, as although vascular structure in the sciatic nerve and spinal cord is normal, baseline neural blood flow is reduced by 44%. In addition to vascular development, however, VEGF plays a central role in the development of the nervous system, and may be required for survival of adult neurones [2]. Disruption of these functions may determine the development of neuronal degeneration in VEGF^δ/δ ^mice.

VEGF has neurotrophic effects *in vitro *in a wide range of culture conditions, promoting cell survival and neurite outgrowth, via its tyrosine kinase receptor, VEGFR2 [3]. *In vivo*, VEGF administration prolongs survival in the SOD1 model of ALS [4]. This is likely to be a direct neuroprotective effect, as SOD1 mice crossed with mice with neuronal overexpression of VEGFR2 also exhibit delayed disease progression [5]. In the developing nervous system, VEGF released by neuroblasts and glia induces the ingrowth of capillaries, and deletion of a single copy of VEGF is lethal [6,7]. VEGF stimulates neurogenesis directly [8], and via endothelial cell proliferation, to form vascular niches in which neurogenesis is stimulated by endothelial-derived BDNF [9,10]. Further interaction between vascular and neuronal development is seen in the closely aligned growth of blood vessels and peripheral nerves, directed by attractive and repulsive cues on the growth cone [11]. The VEGF co-receptor, Neuropilin-1 mediates both repulsive signals of the semaphorin family, and attractant signals of VEGF [11,12]. Thus VEGF, with its angiogenic and neurotrophic actions via VEGFR2, and shared receptor with the semaphorin axon guidance factors, may be a key player in the parallel development of vascular and nervous systems.

A recent meta-analysis of association studies of VEGF polymorphisms with ALS showed an increased risk of ALS in male patients with the -2578AA genotype, which lowers VEGF expression[13]. We have previously shown that levels of expression of VEGF and its main agonist receptor are reduced in the spinal cord of patients with ALS[14]. This study aims to clarify the molecular mechanisms of neurodegeneration in the VEGF^δ/δ ^mouse, by determination of the transcriptional profile of isolated spinal motor neurones in the transgenic mouse, compared to its wild type littermate. Understanding the role of VEGF in the survival and death of motor neurones in this mouse model of ALS may have implications for the human disease.

We report that adult VEGF^δ/δ ^mice show reduction in expression, from symptom onset, of genes involved in nervous system development, particularly in axonogenesis and synapse formation, and that axon outgrowth is reduced in motor neurone cultures derived from VEGF^δ/δ ^mice. These changes suggest a role for VEGF in the maintenance of neuronal circuitry, disruption of which may result in a dying-back axonopathy.

## Methods

### Experimental animals

Adult female VEGF^δ/δ ^mice and wild type littermates (Vesalius Research Institute, Leuven, Belgium) were used in this study. VEGF^δ/δ ^mice, on a Swiss/129 background, had homozygous deletion of the HIF-1 response element (hypoxia response element) in the promoter region of the VEGF gene, and were generated as previously described [1]. All mice were housed in conventional facilities with a 12 h light/dark cycle with access to food at libitum. The local animal ethical committee, the Ethische Commissie Dierproeven (ECD) at the Catholic University Leuven, Belgium, approved the VEGF^δ/δ ^mouse experiments.

### Tissue collection

Three female VEGF^δ/δ ^transgenic mice, and three gender-matched wild-type littermate controls were sacrificed at 3 months (pre-symptomatic), 5 months (onset of symptoms) and 14 months (late stage disease) of age, by overdose of isoflurane inhalational anaesthetic. Post mortem, animals were perfused by intracardiac injection of 15 ml sterile phosphate buffer with 30% sucrose, and the CNS was dissected and frozen in Cryo-M-Bed embedding compound (Bright, Huntingdon, UK). The procedure of sucrose perfusion and dissection of the spinal cord was conducted rapidly, within a maximum of ten minutes from terminal anaesthesia to snap freezing of tissue, to ensure optimal preservation of RNA quality. Lumbar spinal cord sections (10 μm) were fixed in 70% ethanol, washed in DEPC-treated water, and stained for 1 minute in a solution of 0.1% w/v Toluidine Blue in 0.1 M sodium phosphate. They were then washed and dehydrated through graded ethanol concentrations (70, 90 and 100%), and xylene.

### Laser capture microdissection, RNA isolation and amplification

Spinal motor neurones, identified by staining, anatomical location, size and morphology, were isolated on Capsure Macro LCM caps using the Arcturus PixCell II laser capture microdissection system (Arcturus Bioscience, Mountain View, CA). Approximately 1500 motor neurones were dissected from each spinal cord, and >50 ng RNA extracted using the PicoPure™ RNA isolation kit (Arcturus), according to manufacturer's instructions. RNA amplification was carried out using a linear amplification process in 2 cycles, with the GeneChip two cycle target labelling and control kit (Affymetrix, Santa Clara, USA), and MEGAscript^® ^T7 kit (Ambion, Austin, USA). The linear amplification technique has been shown to generate highly reproducible gene expression profiles from small starting quantities of RNA [15]. This procedure produced 50-100 μg of biotin labelled antisense RNA for each sample, the quality and quantity of which was assayed using the Agilent bioanalyser and Nanodrop™ 1000 spectrophotometer (Thermo Scientific, Wilmington, USA).

### Quality control parameters

**Figure 1:**
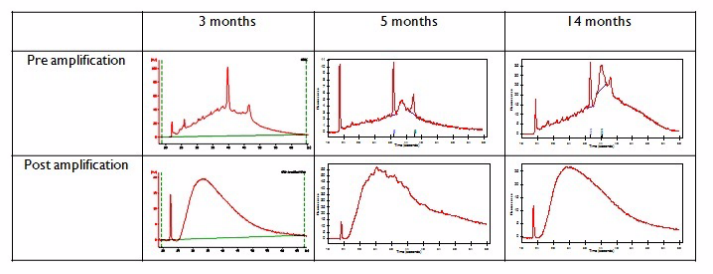
**Representative bioanalyser traces of RNA samples pre- and post-amplification at 3 months, 5 months and 14 months**.

At each stage of extraction, amplification and microarray analysis, we carried out quality control (QC) measures, according to Affymetrix protocols, to ensure that RNA was of sufficient quality and was matched between samples. Where QC outliers were identified, these samples were excluded from the analysis. We used visual interpretation of RNA profiles from bioanalyser traces of extracted and amplified RNA to determine RNA quality, as the 28S:18S ratio has been shown to have only weak correlation with gene expression levels in downstream experiments[16]. The RNA profiles obtained from laser captured material in this study were comparable to profiles with modest RNA degradation, that has been shown to have little effect on the results of microarray analysis [17,18] (Figure 1) Following amplification, the most frequent length of RNA amplicons, at 500-1000 kB, was consistent with Affymetrix protocols. Affymetrix and Bioconductor software was used to generate, from microarray data, the QC measures of average background, signal intensity, percent present calls, and RNA degradation plots, to ensure that samples were comparable in all parameters.

### Affymetrix GeneChip processing

15 μg amplified cRNA was fragmented by heating to 94°C for 20 minutes, and spiked hybridization controls were added. Each sample was hybridized to one mouse 430A2 GeneChip (Affymetrix) according to manufacturer's protocols. Following overnight hybridization at 42°C, GeneChips underwent stringency washes in a Fluidics Station 400, then were scanned in the GeneChip Scanner 3000 to detect fluorescent hybridization signals. These were analysed by the Affymetrix GeneChip Operating System (GCOS) to generate an overall hybridization signal for each transcript from 11 representative perfect match and mismatch probe pairs.

### Microarray data analysis

CEL files generated by GCOS were imported for further analysis into Array Assist software (Stratagene, La Jolla, USA), where probe level analysis was carried out using the GC Robust Multichip Average (GC-RMA) algorithm. Following GCRMA processing, data was filtered to remove those genes whose expression was at or around the background signal level of the chip. Any probe set that returned a signal of <50 on more than 3 chips at each time point was excluded from further analysis. This signal filter did not remove genes that were only expressed in one experimental group. Differential gene expression was determined using an unpaired t-test, to generate a list of genes that were significantly differentially expressed between transgenic mice and their wild type littermates, at each time point.

### Gene ontology analysis

Gene Ontology (GO) terms that reflect the function of the corresponding genes are assigned to each probe set by Affymetrix software. This GO information was used to determine which functionally related groups of genes were over-represented amongst significantly differentially expressed genes at each time point. The frequencies of GO terms represented in the significant gene lists were compared to a denominator list of genes that can be expressed by motor neurones in health or disease. GO analysis was carried out without regard for fold change, in order to limit type II errors and in order to detect more subtle changes in gene expression, for example in transcriptional regulators, which may be biologically significant. The denominator list was generated from the array data for each time point, by extracting those genes whose representative probe sets returned a signal of >50 in 3 or more chips. Statistical analysis of GO term enrichment was carried out using DAVID software (NIAID/NIH; http://david.abcc.ncifcrf.gov/summary.jsp, [19]. Literature review was also used to identify significantly differentially expressed genes with functions relating to those identified as enriched by DAVID analysis.

### Verification of microarray results by Quantitative rt-PCR (QPCR)

A proportion of genes identified as significantly differentially expressed were selected for verification by QPCR, on the basis of robust microarray data confirming differential gene expression, involvement in a biological process identified as enriched by GO analysis, or a known function in neurodegeneration. Verification addresses the possibility of false positive microarray signals, due to cross-hybridization with related genes, concern about the accuracy of array probe sets, and uncertainty about the hybridization kinetics of multiple reactions occurring on the miniature scale of an array chip. RNA was extracted from 1000-1500 cells, isolated by laser capture microdissection from the lumbar spinal cord of the population of mice used in the microarray experiment and, where available, a second population of transgenic mice with wild-type littermates. RNA was extracted, quantified as previously described, and reverse transcribed to cDNA using Superscript II reverse transcriptase, according to manufacturer's protocol (Invitrogen, San Diego, CA). Primers used in for verification are shown in Table 1. QPCR was performed using 12.5 ng cDNA, 1×SYBR Green PCR master mix (Applied Biosystems, Foster City, CA), and forward and reverse primers at optimized concentrations, to a total volume of 20 μl. After an initial denaturation at 95°C for 10 mins, templates were amplified by 40 cycles of 95°C for 15 secs and 60°C for 1 minute, on an MX3000P Real-Time PCR system (Stratagene). A dissociation curve was then generated to ensure amplification of a single product, and absence of primer dimers. For each primer pair, a standard curve was generated to determine the efficiency of the PCR reaction over a range of template concentrations from 0.3 ng/μl to 25 ng/μl, using cDNA synthesized from mouse universal RNA. The efficiency for each set of primers was 100+/-10%, such that gene expression values, normalized to ß-actin expression, could be determined using the ddCt calculation (ABI PRISM 7700 Sequence Detection System protocol; Applied Biosystems). An unpaired t-test was used to determine the statistical significance of any differences in gene expression. β-actin hybridization signals determined by microarray analysis confirmed that there was no significant difference in β-actin expression between wild type and transgenic mice. To determine the effect of the choice of the normalizing gene on the verification of microarray results by QPCR, the expression of four genes was also determined using two alternative normalizing genes, HspB8 and Nme1. These were identified by microarray analysis as having the most consistent levels of expression in spinal motor neurones at each time point, with the lowest coefficient of variation of hybridization signals.

**Table 1:** Primer pairs used for QPCR experiments

Gene symbol	Forward sequence	Reverse Sequence	Optimal primer conc F/R (fmol)
**3' Actin**	GCTCTGGCTCCTAGCACCAT	AGCCACCGATCCACACAGAGT	300/300
**HspB8**	GGGCCTGCTCATCATCGA	GAGGAAGCTCGTTGTTGAAGCT	300/300
**Nme1**	CGCAGAACTGGATCTATGAGTGA	CCCCTGCCTGTGAGAACAA	300/300
**Zfp101**	GGATGAAATCCTGTTCCCATACAT	TGTCCTGGATTAGATACTGTATTTTGATA	900/900
**Fos**	ATTGTCGAGGTGGTCTGAATGTT	AACGTTTTCATGGAAAACTGTTAATG	300/300
**Rln**	AAGCACTCGCAAACAAAATTACAT	CCTAAGCGACCTTCGTCTTCTG	900/900
**HspA5**	CCTCAGAGTGGAGTTGAAAATGCTA	GACCCCAAGACATGTGAGCAA	300/900
**Ldlr**	ACCTGGCTCGGTTTTCATTCT	AGAGTATCACCCCAGCCTAACCT	900/900
**Scd1**	GACCAGTCAAAGTGCAAGACTACCTA	AAGGTTTCCTGCAATGGTTTTC	300/300
**Nrp1**	CGGTAACAACAGGAATCATGTACAA	TTACCCAAATGAAACCAAGAGAAGA	300/300
**Mtap1B**	CCGTTGCACCTTTCGTAGCT	AGCCAATGCAAGACAAAGGAA	100/100
**Alcam**	GGACACATATCTTGCCCAATCAG	ATCCTATGGTGCTCCTAACTCTCAA	300/900
**HNRPDL**	TTGTAAAAGACTTTGTACTCTAGATCAGAGA	TGGCAGCTATATAGACTTCCAGAGA	300/900
**Tnrc6a**	ATGTTGGACACCGTAACCTAAGC	TATGGACATCAACACACACCGAAT	300/300
**VEGF**	ATGCTCCCCGGGGTGTAT	CATAGGAGTCCTTCTGACCCATTC	600/600

### Neural VEGF quantitation

Ten 10 μm sections of cervical cord were taken from each mouse used in the microarray study. RNA was extracted using the PicoPure™ RNA isolation kit (Arcturus) and reverse transcribed to cDNA with Superscript II reverse transcriptase (Invitrogen), according to manufacturer's protocols. Neural VEGF expression was assessed by QPCR, as described above, normalized to the expression of ß-actin. Primer sequences and concentrations are shown in Table 1. VEGF expression was compared between VEGF^δ/δ ^and wild-type mice using an unpaired t test.

### Isolation of embryonic motor neurons and quantification of axonal outgrowth

Cultures of spinal motor neurons from E12.5 mice (VEGF^wt/wt^, n = 8; VEGF^δ/wt^, n = 14; and VEGF^δ/δ^, n = 4) were prepared by a panning technique using a monoclonal anti-p75NTR antibody (Millipore Bioscience Research Reagents)[20]. The ventrolateral parts of individual lumbar spinal cords were dissected and transferred to HBSS containing 10 mM 2-mercaptoethanol. After treatment with trypsin (0.05%, 10 min), single cell suspensions were generated by titration. The cells were plated on an anti-p75NTR coated culture dish (24 well; Greiner) and left at room temperature for 30 min. The individual wells were subsequently washed with HBSS (three times), and the attaching cells were then isolated from the plate with depolarizing saline (0.8% NaCl, 35 mM KCl, and 1 mM 2-mercaptoethanol) and plated at a density of 1500 cells per well on laminin-coated coverslips in Greiner four-well culture dishes. Motor neurons from single embryos were cultured for 7 days in quadruplicates in the presence of BDNF and CNTF (1 ng/ml each, 2000 cells/well initially). Surviving neurons were counted on day 0, 1 and day 7. Initial counting of plated cells was done when all cells were attached to the culture dish at 4 h after plating. At day 7, wells were fixed with fresh 4% paraformaldehyde in phosphate buffer and subjected to immunocytochemistry. Two coverslips with motor neurons of each embryo were stained with antibodies against MAP2 and P-Tau to distinguish between axons and dendrites. Axon lengths of VEGF^wt/wt ^(n = 202), VEGF^δ/wt^, (n = 406) and VEGF^δ/δ ^(n = 122) neurones were measured using the Leica Confocal Software (Leica, Germany), and compared using a t test.

## Results

### Neural VEGF expression

VEGF^δ/δ ^mice used in this study showed a reduction in expression of VEGF mRNA in the cervical spinal cord of 33% compared to their wild type littermates (data not shown). A similar reduction in baseline spinal VEGF protein expression in the spinal cord, although not of mRNA expression, was described by Oosthuyse *et al *[1].

### General features of differential gene expression between VEGF^δ/δ ^and wild type mice

**Figure 2:**
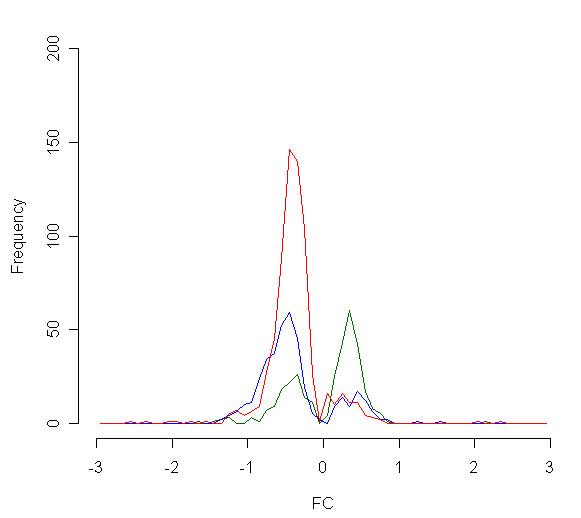
**Histogram of fold change values of significantly differentially expressed genes at 3 months (green), 5 months (blue) and late stage (red)**. Upregulated genes are plotted on the positive axis, downregulated genes on the negative axis.

Between 15 and 18% of probe sets at each time point passed the signal filter, and their corresponding genes were considered expressed by motor neurones. Statistical analysis of these genes showed that 324 genes were significantly differentially expressed (at p < 0.05 level) between VEGF^δ/δ ^and wild type mice at 3 months, 382 genes at 5 months, and 689 genes at late stage. The total numbers of genes which are upregulated and downregulated, with three different p values (0.05, 0.01 and 0.001) are given for each time point in Table 2. Prior to symptom onset, the majority of differentially expressed genes were upregulated. At disease onset, this pattern had reversed, and the majority of genes were significantly downregulated. At late stage disease there was marked transcriptional downregulation, shown in Figure 2. Fold changes between VEGF^δ/δ ^mice and their littermates were, in general, smaller than those seen in parallel experimental design using the mutant SOD1 model of ALS [21].

**Table 2:** Numbers of significantly differentially expressed genes at each time point

3 months		ALL FC		>1.5		>2	
**PROBE SETS THAT PASS SIGNAL FILTER**		UP	DOWN	UP	DOWN	UP	DOWN
6563	p < 0.05	207	117	16	27	2	6
	p < 0.01	26	28	6	9	1	2
	p < 0.001	3	7	2	2	1	1

**5 months**		**ALL FC**		**>1.5**		**>2**	

**PROBE SETS THAT PASS SIGNAL FILTER**		UP	DOWN	UP	DOWN	UP	DOWN
7236	p < 0.05	73	311	10	138	2	23
	p < 0.01	10	60	0	31	0	6
	p < 0.001	1	5	0	3	0	1

**14 months**		**ALL FC**		**>1.5**		**>2**	

**PROBE SETS THAT PASS SIGNAL FILTER**		UP	DOWN	UP	DOWN	UP	DOWN
8200	p < 0.05	75	614	7	120	2	22
	p < 0.01	10	146	2	57	0	12
	p < 0.001	1	22	0	9	0	2

### Gene ontology analysis

Gene ontology (GO) terms which were significantly enriched (at level p < 0.05) amongst genes significantly differentially expressed between transgenic and wild type mice were identified for each time point. Over-represented GO terms at each time point are given in Tables 3, 4 and 5. Four GO clusters were identified: Ontology terms concerned with mitochondrial function and energy production, with steroid metabolism, with axonogenesis and nervous system development, and with gene expression and RNA metabolism. There is overlap between GO terms enriched at 3 months and 5 months, and between 5 months and late stage disease, suggesting that the processes represented by these terms are affected in transgenic mice in a sequential manner, as disease progresses.

**Table 3:** GO terms significantly enriched amongst differentially expressed genes at 3 months

Gene ontology term	No. of genes	Fold Enrichment	p value
**Energy production**			
tricarboxylic acid cycle intermediate metabolic process	4	9.8	0.0066
generation of precursor metabolites and energy	18	1.9	0.0128
Carbohydrate metabolic process	14	1.8	0.0454

**Other**			
cardiac muscle cell differentiation	3	12.7	0.0212
striated muscle cell differentiation	4	6.2	0.0246
cardiac cell differentiation	3	9.8	0.0348
response to temperature stimulus	4	4.9	0.0454

**Table 4:** GO terms significantly enriched amongst differentially expressed genes at 5 months

Gene ontology term	No. of genes	Fold Enrichment	p value
**Nervous system development**			
axon extension	6	8.1	0.0006
cell part morphogenesis	18	2.4	0.0010
cell projection organization and biogenesis	18	2.4	0.0010
cell projection morphogenesis	18	2.4	0.0010
regulation of axon extension	5	10.2	0.0010
regulation of axonogenesis	6	5.6	0.0035
regulation of neurogenesis	7	4.5	0.0040
cellular morphogenesis during differentiation	13	2.5	0.0055
positive regulation of cell adhesion	4	8.9	0.0087
axonogenesis	11	2.6	0.0090
neurite development	13	2.3	0.0093
nervous system development	27	1.7	0.0105
cell morphogenesis	23	1.7	0.0135
cellular structure morphogenesis	23	1.7	0.0135
neurite morphogenesis	11	2.4	0.0175
neuron morphogenesis during differentiation	11	2.4	0.0175
neuron development	13	2.1	0.0183
microtubule-based process	12	2.1	0.0249
negative regulation of axon extension	3	10.5	0.0304
neuron differentiation	14	1.9	0.0346
negative regulation of neurogenesis	4	5.4	0.0348
Neurogenesis	16	1.7	0.0403
cell migration	13	1.9	0.0413
generation of neurons	15	1.8	0.0457
negative regulation of axonogenesis	3	8.1	0.0493

**Cholesterol metabolism**			
cholesterol metabolic process	7	3.9	0.0083
steroid biosynthetic process	8	4.3	0.0021
sterol biosynthetic process	6	5.9	0.0030
sterol metabolic process	8	3.9	0.0039
steroid metabolic process	10	2.9	0.0064
cholesterol biosynthetic process	5	6.1	0.0078

**RNA metabolism**			
nuclear mRNA splicing, via spliceosome	7	3.4	0.0153
RNA splicing, via transesterification reactions	7	3.4	0.0153

**Energy production**			
generation of precursor metabolites and energy	20	1.7	0.0282

**Other**			
protein import	8	3.3	0.0106
leukocyte migration	4	8.1	0.0112
protein import into nucleus	7	3.6	0.0115
leukocyte migration	4	8.1	0.0112
protein import into nucleus	7	3.6	0.0115
nuclear import	7	3.5	0.0140
nucleocytoplasmic transport	9	2.5	0.0249
feeding behaviour	4	6.1	0.0253
nuclear transport	9	2.5	0.0265
alcohol metabolic process	14	1.9	0.0296

**Table 5:** GO terms significantly enriched amongst differentially expressed genes at 14 months

Gene ontology term	No. of genes	Fold Enrichment	p value
**RNA metabolism and regulation of gene expression**			
protein-RNA complex assembly	11	2.5	0.011
mRNA metabolic process	23	1.7	0.012
mRNA processing	21	1.8	0.014
gene expression	136	1.2	0.015
RNA splicing	18	1.8	0.018
Regulation of gene expression	98	1.2	0.024
Regulation of transcription	91	1.2	0.032
regulation of nucleoside, nucleotide and nucleic acid metabolic process	92	1.2	0.040
transcription	93	1.2	0.047
negative regulation of gene expression, epigenetic	4	4.7	0.048

**Nervous system development**			
neurite morphogenesis	14	2.0	0.018
neuron morphogenesis during differentiation	14	2.0	0.018
axonogenesis	13	2.1	0.019
neurite development	15	1.8	0.037
cellular morphogenesis during differentiation	14	1.8	0.040
neuron development	16	1.7	0.045

**Other**			
nuclear export	8	4.6	0.001
protein export from nucleus	5	6.9	0.004
negative regulation of metabolic process	23	1.6	0.029
phosphatidylinositol metabolic process	4	5.5	0.032
nucleocytoplasmic transport	11	2.1	0.034
macromolecule biosynthetic process	41	1.4	0.034
nuclear transport	11	2.1	0.036
negative regulation of cellular metabolic process	20	1.6	0.046
phospholipid metabolic process	11	2.0	0.047
heme metabolic process	4	4.7	0.048

### Mitochondrial function and energy production

**Figure 3:**
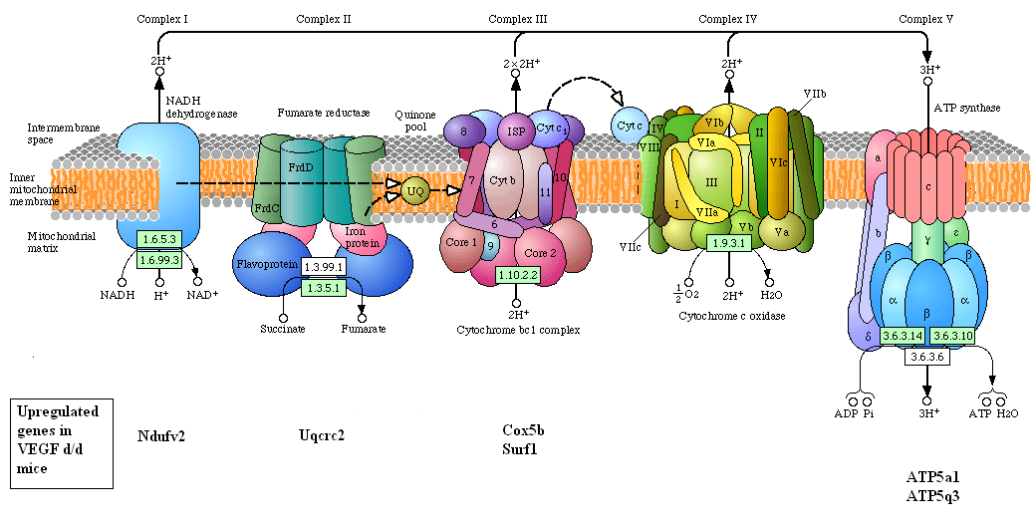
**Diagrammatic representation of the components of the mitochondrial electron transport chain, and the constituent proteins encoded by genes significantly upregulated in VEGF^δ/δ ^mice**.

In presymptomatic mice, fewer transcriptional changes were seen in transgenic mice, compared to their wild-type littermates than at the other time points of disease. The most significantly over-represented GO terms related to cellular energy production (TCA cycle metabolism, generation of precursor metabolites and energy and carbohydrate metabolism). The differentially expressed genes included several genes encoding TCA cycle enzymes and components of the electron transport chain (Table 6). Three genes encoding TCA cycle enzymes, *Mdh2*, *Ldh2 *and *Oxct1*, are upregulated, while *Ldh1*, an isoform that favours glycolysis over oxidative metabolism [22], is downregulated. In the electron transport chain, genes that produce components of 4 out of the 5 complexes, and *Pcdc8*, which is required for mitochondrial oxidative phosphorylation [23], show upregulation (Figure 3). These gene expression changes would be consistent with a small but significant increase in oxidative metabolism in neurones of VEGF^δ/δ ^mice in the early stages of disease. Concomitant with this increase, there was upregulation of the free radical scavenging enzymes, *Prdx2 *and *Sod2*.

**Table 6:** Differentially regulated genes involved in cellular energy production at 3 months

Probe ID	Gene title	Symbol	p value	FC	Regn
**Electron transport chain**					
1438159	NADH dehydrogenase (ubiquinone) flavoprotein 2	Ndufv2	0.0330	1.15	up
1435757	Ubiquinol cytochrome C reductase core protein 2	Uqcrc2	0.0066	1.21	up
1456588	Cytochrome c oxidase, subunit Vb	Cox5b	0.0460	1.16	up
1450561	Surfeit gene 1	Surf1	0.0187	1.16	up
1449710	ATP synthase H+ transporting, mitochondrial F1 complex, α subunit, isoform 1	Atp5a1	0.0216	1.09	up
1454661	ATP synthase H+ transporting, mitochondrial F0 complex, subunit c, isoform 3	Atp5g3	0.0309	1.06	up
1418127	Programmed cell death 8 (Apoptosis inducing factor)	Pcdc8	0.0185	1.26	up

**TCA cycle**					
1419737	Lactate dehydrogenase 1, A chain	Ldh1	0.0077	1.86	down
1433984	Malate dehydrogenase 2, NAD	Mdh2	0.0188	1.16	up
1455235	Lactate dehydrogenase 2, B chain	Ldh2	0.0216	1.12	up
1436750	3-oxoacid CoA transferase1	Oxct1	0.0441	1.30	up

**Free radical scavenging**					
1430979	Peroxiredoxin 2	Prdx2	0.0397	1.27	up
1444531	Superoxide dismutase 2, mitochondrial	Sod2	0.0417	1.20	up

### Cholesterol metabolism

**Figure 4:**
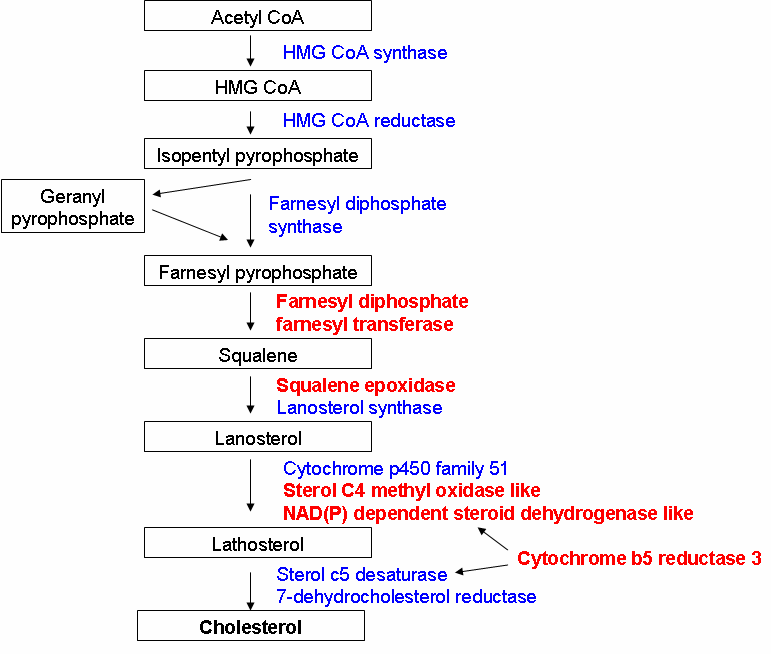
**Schematic representation of the cholesterol biosynthesis pathway, with genes that are differentially regulated in VEGF^δ/δ ^mice at 5 months highlighted in red**. Cytochrome B5 reductase is an electron carrier for 5-desaturase and methyl sterol oxidase [119]

At disease onset, there was enrichment of genes assigned GO terms relating to steroid metabolism, all of which were downregulated (Table 7). These included five genes that catalyse reactions in the final committed pathway to cholesterol synthesis pathway (Figure 4). *Sqle *is a rate limiting step in this pathway [24]. *Prkaa2 *is a catalytic subunit of AMPK, which regulates HMG CoA reductase activity. *Ldlr *and *Sorl1 *have similar functions in binding LDL, the major cholesterol carrying lipoprotein of plasma, and transporting it into cells. *Hsd17b7 *catalyses the synthesis of steroid hormones from cholesterol, while *Stard4 *promotes transport of cholesterol across the mitochondrial membrane, and stimulates steroidogenesis [25]. *Nr3c1 *is the glucocorticoid receptor.

**Table 7:** Differentially expressed genes in the category of 'Steroid metabolism' at 5 months

Probe ID	Gene Title	Symbol	p value	FC	Regn
1416222	NAD(P) dependent steroid dehydrogenase-like	Nsdhl	0.0372	1.77	down
1421821	low density lipoprotein receptor	Ldlr	0.0172	2.13	down
1422185	cytochrome b5 reductase 3	Cyb5r3	0.0248	2.24	down
1423078	sterol-C4-methyl oxidase-like	Sc4mol	0.0161	2.10	down
1429240	StAR-related lipid transfer (START) domain containing 4	Stard4	0.0044	1.83	down
1438322	farnesyl diphosphate farnesyl transferase 1	Fdft1	0.0442	1.56	down
1448130	farnesyl diphosphate farnesyl transferase 1	Fdft1	0.0323	1.86	down
1457248	hydroxysteroid (17-beta) dehydrogenase 7	Hsd17b7	0.0250	1.74	down
1460390	sortilin-related receptor, LDLR class A	Sorl1	0.0499	1.33	down
1415993	squalene epoxidase	Sqle	0.0204	1.97	down

### Nervous system development

**Figure 5:**
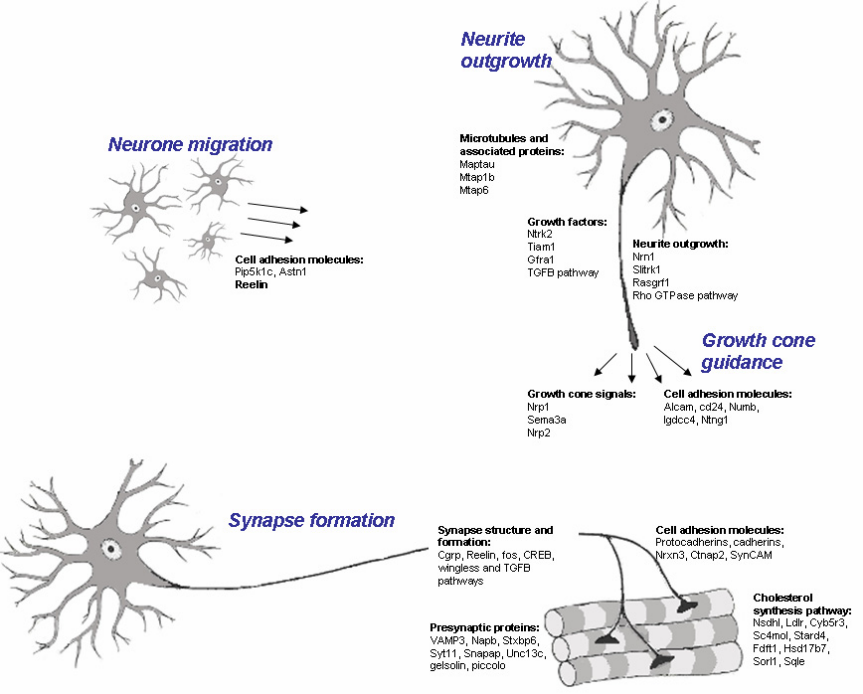
**Schematic representation of genes involved in neuronal migration, neurite outgrowth and formation and maintenance of the neuromuscular junction, which are all downregulated in the VEGF transgenic mouse**.

At all stages of disease, there was differential expression of genes involved in nervous system development, with prominent enrichment of GO terms relating to nervous system development in the significant gene lists at both 5 months and 14 months. Genes with functions relating to nervous system development at these time points, the majority of which are downregulated, are shown in Table 8 and Figure 5. Several are identified by more than one probe set or at more than one time point.

**Table 8:** Differentially expressed genes in the category of 'Nervous system development'

Probe ID	Gene Title	Symbol	p value	FC	Regn	Time point
**Neurite outgrowth**						
1428393	neuritin 1	Nrn1	0.0090	3.82	down	14 months
1428089	SLIT and NTRK-like family, member 1	Slitrk1	0.0156	1.35	down	5 months
1416666	serine peptidase inhibitor, clade E, member 2	Serpine2	0.0414	1.65	down	5 months
1437308	coagulation factor II (thrombin) receptor	F2r	0.0346	1.59	up	3 months
1437308	coagulation factor II (thrombin) receptor	F2r	0.0286	1.22	up	5 months
1455252	tuberous sclerosis 1	Tsc1	0.0073	1.37	up	5 months
1422600	RAS protein-specific guanine nucleotide-releasing factor 1	Rasgrf1	0.0011	1.63	down	14 months
1435614	RAS protein-specific guanine nucleotide-releasing factor 1	Rasgrf1	0.0002	1.44	down	14 months
1455027	RUN and FYVE domain containing 2	Rufy3	0.0360	1.43	down	5 months
1424402	RUN and FYVE domain containing 2	Rufy3	0.0382	1.32	down	5 months
1452342	amyloid beta precursor protein-binding, family B, member 2	Apbb2	0.0415	1.21	down	14 months
1449439	Kruppel-like factor 7 (ubiquitous)	Klf7	0.0008	1.62	down	5 months

**Growth factor signalling pathways**						
1420838	neurotrophic tyrosine kinase, receptor, type 2	Ntrk2	0.0067	1.29	down	5 months
1418057	T-cell lymphoma invasion and metastasis 1	Tiam1	0.0006	1.66	down	5 months
1418057	T-cell lymphoma invasion and metastasis 1	Tiam1	0.0031	1.43	down	14 months
1439015	glial cell line derived neurotrophic factor receptor alpha 1	Gfra1	0.0454	1.95	down	5 months
1455018	lemur tyrosine kinase 2	Lmtk2	0.0107	1.32	down	14 months

**TGFß signalling pathway**						
1450923	transforming growth factor, beta 2	Tgfb2	0.0397	1.47	down	5 months
1450839	DNA segment, human D4S114	D0H4S114	0.0093	2.00	down	3 months
1450839	DNA segment, human D4S114	D0H4S114	0.0190	1.98	down	5 months
1450839	DNA segment, human D4S114	D0H4S114	0.0025	1.50	down	14 months
1422487	MAD homolog 4 (Drosophila)	Smad4	0.0368	1.41	down	5 months
1452143	spectrin beta 2	Spnb2	0.0023	1.21	down	5 months
1425116	spectrin beta 4	Spnb4	0.0463	1.14	up	14 months

**Rho-GTPase pathway**						
1416329	cytoplasmic FMR1 interacting protein 1	Cyfip1	0.0158	1.36	down	5 months
1448600	vav 3 oncogene	Vav3	0.0304	1.32	down	5 months

**Microtubule-based processes**						
1455719	tubulin, beta 5	Tubb5	0.0034	1.60	down	5 months
1421851	microtubule-associated protein 1 B	Mtap1b	0.0142	2.10	down	5 months
1457316	microtubule-associated protein 6	Mtap6	0.0105	1.75	down	5 months
1424719	microtubule-associated protein tau	Mapt	0.0482	1.50	down	5 months
1417005	kinesin 2	Kns2	0.0484	1.17	down	5 months
1433926	dynein, cytoplasmic, light intermediate chain 2	Dnclic2	0.0121	1.36	down	5 months
1437875	bicaudal D homolog 2	Bicd2	0.0236	1.45	down	5 months
1451630	tubulin tyrosine ligase	Ttl	0.0016	1.45	down	5 months
1428282	tubulin-specific chaperone e	Tbce	0.0348	1.24	down	14 months
1423626	dystonin	Dst	0.0358	1.24	down	5 months

**Axon guidance**						
1418084	neuropilin 1	Nrp1	0.0092	2.02	down	5 months
1448944	neuropilin 1	Nrp1	0.0151	1.58	down	5 months
1456778	neuropilin 2	Nrp2	0.0020	1.28	down	5 months
1420416	semaphorin 3A	Sema3a	0.0010	1.35	down	5 months
1420416	semaphorin 3A	Sema3a	0.0341	1.26	down	14 months
1415877	dihydropyrimidinase-like 3	Dpysl3	0.0461	2.25	down	5 months
1450863	double cortin and calcium/calmodulin-dependent protein kinase-like 1	Dcamkl1	0.0364	1.29	down	5 months

**Synapse formation and plasticity**						
1427898	ring finger protein (C3H2C3 type) 6	Rnf6	0.0182	1.59	down	5 months
1452004	calcitonin-related polypeptide, alpha	Calca	0.0283	1.26	down	5 months
1422639	calcitonin-related polypeptide, beta	Calcb	0.0389	1.78	down	5 months
1449465	reelin	Reln	0.0006	2.38	down	3 months
1423100	FBJ osteosarcoma oncogene	Fos	0.0313	2.32	down	3 months
1423100	FBJ osteosarcoma oncogene	Fos	0.0123	2.17	down	5 months
1452901	cAMP responsive element binding protein 1	Creb1	0.0148	1.44	down	14 months

**Wingless signalling pathway**						
1437301	dishevelled, dsh homolog 1 (Drosophila)	Dvl1	0.0166	1.38	down	14 months
1434439	glycogen synthase kinase 3 beta	Gsk3b	0.0023	1.23	down	14 months
1437351	CXXC finger 4	Cxxc4	0.0462	1.37	down	5 months
1437351	CXXC finger 4	Cxxc4	0.0341	1.30	down	14 months

**Cell adhesion molecules**						
1437466	activated leukocyte cell adhesion molecule	Alcam	0.0329	1.94	down	5 months
1437467	activated leukocyte cell adhesion molecule	Alcam	0.0233	1.60	down	5 months
1437466	activated leukocyte cell adhesion molecule	Alcam	0.0400	1.65	down	14 months
1426301	activated leukocyte cell adhesion molecule	Alcam	0.0045	1.51	down	14 months
1437467	activated leukocyte cell adhesion molecule	Alcam	0.0414	1.50	down	14 months
1416034	CD24a antigen	Cd24a	0.0165	1.96	down	5 months
1416891	numb gene homolog (Drosophila)	Numb	0.0394	1.45	down	14 months
1424954	phosphatidylinositol-4-phosphate 5-kinase, type 1 gamma	Pip5k1c	0.0166	1.14	down	14 months
1418615	astrotactin 1	Astn1	0.0461	1.65	down	5 months
1416474	immunoglobulin superfamily, DCC subclass, member 4	Igdcc4	0.0342	1.19	down	5 months
1449286	netrin G1	Ntng1	0.0119	1.87	down	14 months
1442659	protocadherin 9	Pcdh9	0.0166	1.64	down	14 months
1420429	protocadherin beta 3	Pcdhb3	0.0365	1.89	down	14 months
1425092	cadherin 10	Cdh10	0.0208	1.39	down	14 months
1433788	neurexin III	Nrxn3	0.0021	1.78	down	14 months
1422798	contactin associated protein-like 2	Cntnap2	0.0077	1.37	down	5 months
1417378	synaptic cell adhesion molecule	Syncam	0.0400	1.40	down	14 months
1417377	synaptic cell adhesion molecule	Syncam	0.0191	1.38	down	14 months
1422445	integrin alpha 6	Itga6	0.0221	1.50	down	5 months

**Others**						
1457015	Neurofilament 3, medium	Nef3	0.0209	1.40	up	14 months
1416533	EGL nine homolog 2	Egln2	0.0203	1.46	down	5 months
1420475	myotrophin	Mtpn	0.0120	1.67	down	5 months
1417133	peripheral myelin protein	Pmp22	0.0159	1.89	down	5 months
1449353	wild-type p53-induced gene 1	Wig1	0.0249	1.57	down	5 months
1417624	Ngfi-A binding protein 1	Nab1	0.0119	1.44	down	5 months

There is downregulation in VEGF^δ/δ ^mice of genes that promote neurite outgrowth, such as *Nrn1 *[26], *Slitrk1 *[27], *RasGrf1*[28] and *Serpine2*, which is an inhibitor of thrombin [29]. Thrombin causes neurite retraction and neuronal death via its receptor *F2r*, which is upregulated at 3 and 5 months [30]. *Tsc1*, which inhibits axon growth, is also upregulated at both time points [31]. Growth factors such and NGF, BDNF, GDNF and TGFß2 promote neuronal differentiation and outgrowth [32,33], There is downregulation in motor neurones of VEGF^δ/δ ^mice of the BDNF receptor, *Ntrk2*, and its downstream mediator, *Tiam1 *[34]; the GDNF receptor, *Gfra1*; several components of the TGFß2 signaling pathway; and *Lmtk2*, a mediator of NGF signalling [35]. *Rufy3 *is implicated in the formation of a single axon, to determine neuronal polarity [36]. *Apbb2 *interacts with amyloid precursor protein, which is required for the maintenance of dendrites and synapses [37]. *KLF7 *is a transcription factor with a key role in neuronal morphogenesis. Null mutations in Klf7 lead to deficits in neuronal outgrowth and axon guidance [38]. The Rho GTPase pathway controls cytoskeletal reorganization to regulate neurone outgrowth, and the maturation and maintenance of dendritic spines. The Rho GTPase, Rac, is activated by *vav3 *[39]. *Cyfip1 *interacts with Rac and null mutations lead to defects in axon growth, guidance and branching, and in the organization of the neuromuscular junction [40].

The GO category 'microtubule-based process' is over-represented at 5 months, with downregulation of genes that are assigned this function. Microtubules are prominent elements of the neuronal cytoskeleton, involved in the growth and maintenance of neurites, and along which motor proteins move. Microtubule associated proteins bind to the tubulin substrate that make up microtubules, and regulate their stability [41]. Tubulin ß5 and three microtubule associated proteins, *Maptau*, *Mtap1b *and *Mtap6*, are downregulated in VEGF^δ/δ ^mice, as are two motor proteins, *kinesin 2 *and *Dnclic2*, and *BICD2 *which binds cargoes to dynein. Disruption of the dynein-dynactin pathway through prevention of BICD2 uncoupling causes motor neurone degeneration in mice [42]. *Ttl *causes post-translational modifications of αtubulin that are essential to neurite extension and normal brain development [43]. *Tbce *is a tubulin chaperone, required for stabilization of neuronal processes, a mutation of which causes the progressive motor neuronopathy of the *pmn *mouse [44]. *Dystonin *is thought to have a role in cytoskeletal cross-linking leading to axonal stability [45].

Differentially expressed genes in the VEGF^δ/δ ^model encode a number of proteins that mediate attractive and repulsive growth cone signals during axonal guidance, including *Nrp1 *and its ligand *sema3A *[46] and *Nrp2 *[47]. Two downregulated genes have regulatory functions in the growth cone tip: *Dpysl3 *and *DCAMKII *[48,49].

Cellular adhesion molecules are important in contact-dependent regulation of axonal growth, and in the control of neuronal migration. Fourteen adhesion molecules and related molecules are downregulated in the VEGF^δ/δ ^mouse. *Alcam *is a member of the immunoglobulin superfamily which has a specific role in the guidance of motor axons and formation of neuromuscular junctions (NMJs) [50]. Both L1 and cd24 interact and cooperate with each other as potent stimulators of neurite outgrowth [51]. *Cd24*, and *Numb*, which mediates endocytosis of the cell-adhesion molecule L1 [52] are downregulated in VEGF^δ/δ ^mice. The DCC (deleted in colon cancer) subgroup of the immunoglobulin superfamily are ligands for netrin, with roles in the migration and guidance of axonal growth cones. *Igdcc4 *is a newly recognized member of this group, which is downregulated at 5 months [53], as is the netrin *Ntng1*[54]. Both *Pip5k1c *and *Astn1 *are required for normal neurone migration [55,56].

The formation of synapses involves guidance of axonal processes towards target cells, target recognition, followed by recruitment of pre- and post-synaptic elements. The synaptic connections between motor neurones and muscle exhibit both functional and anatomical plasticity after maturation, with changes in synaptic strength, and the formation and retraction of neuronal sprouts from synaptic terminals or Nodes of Ranvier. This enables the neuromuscular system to compensate for growth, changes in muscle use, and damage or disease [57]. Adhesion molecules play a central role in the formation and plasticity of synapses, several of which are downregulated in the VEGF^δ/δ ^mouse, including two protocadherins; cadherin 10 [58,59]; two members of the neurexin family, *Nrxn3 *and *Cntnap2*, which bind postsynaptic neuroligins [60,61]; and *SynCAM*, a member of the immunoglobulin superfamily. *Rnf6 *binds to LIM kinase 1, which regulates actin dynamics and is important in determining synaptic structure [62]. Calcitonin-gene-related peptide, of which both polypeptide chains are downregulated in the VEGF^δ/δ ^mouse, is released from motor neurones to stimulate acetylcholine receptor synthesis by muscle, at the NMJ [63]. Reelin is an extracellular matrix protein with a well-recognized function in neuronal migration. More recently, it has been shown to play a role in synaptic plasticity [64], and in the maturation of synaptic contacts during development, by refinement of NMDA-receptor subunit composition [65]. The activation of downstream signalling pathways of reelin is cholesterol-dependent [66]. Fos and Jun form the heterodimer, AP-1, which plays a central role in controlling development, growth, survival and plasticity of neurones. AP-1 has also been shown to positively regulate synapse strength and number, acting upstream of CREB [67]. Both Fos and CREB are downregulated in the VEGF^δ/δ ^mouse.

Proteins of the canonical wingless signalling pathway participate in the assembly of the NMJ, with crucial components being the wingless co-receptor, arrow, Dishevelled and GSK3ß. GSK3ß functions by regulating the structure of the microtubule cytoskeleton, probably via the microtubule-associated protein, MAP1B. Mutations in this pathway cause aberrant NMJ formation, with reduction in number of synaptic boutons [68]. *Dishevelled*, *GSK3ß *and *MAP1B *are downregulated in the VEGF^δ/δ ^model, as is C*XXC4 *which regulates the wnt-dishevelled signalling pathway [69]. TGFß also plays role in the development and functioning of synapses [70]. There is downregulation in the VEGF^δ/δ ^mouse of TGFß2; D0H4S114, which regulates TGF signalling [71]; Smad4 and ß-spectrin at disease onset, which associate in response to TGFß, and are required for the assembly of the NMJ [72,73].

### Pre-synaptic proteins

Concomitant with the downregulation of genes involved in the formation and morphological plasticity of synapses, a reduction was seen in the expression of several genes encoding proteins that comprise the pre-synaptic machinery (Table 9), including several genes involved in the fusion of synaptic vesicles, which is mediated by the SNARE complex of VAMP/synaptobrevin, syntaxin and SNAP25. Both *VAMP3 *and *Nap*b were downregulated in VEGF^δ/δ ^mice, as was the syntaxin-binding protein, *Stxbp6*; *SytI1*, one of a family of calcium-binding proteins that need to be bound to the SNARE complex for pore opening to occur; the SNAP associated protein, *Snapap*, which enhances association of synaptotagmin with the SNARE complex [74]; *Unc13C *which binds to and activates syntaxin [75]; *gelsolin *which disassembles the actin network to liberate synaptic vesicles for release [76]; *piccolo*, which is a scaffolding protein involved in the organization of synaptic active zones, where synaptic vesicles dock and fuse [77]; and synapsins I and II, which modulate neurotransmitter release, possibly by maintaining a pool of synaptic vesicles near to the active zone [78]. PPI controls activity of ion channels and signal transduction enzymes to determine functional synaptic plasticity, and downregulation of several of its regulatory and catalytic subunits was seen, in addition to *Phactr1 *and *2*, which regulate PPI activity [79].

**Table 9:** Genes encoding presynaptic proteins, differentially expressed in VEGF^δ/δ ^mice

Probe ID	Gene name	Symbol	p value	FC	Reg	Time point
1456245	vesicle-associated membrane protein 3	Vamp3	0.0159	1.47	down	14 months
1423173	N-ethylmaleimide sensitive fusion protein ß	Napb	0.0021	1.59	down	14 months
1423172	N-ethylmaleimide sensitive fusion protein ß	Napb	0.0024	1.55	down	14 months
1433788	N-ethylmaleimide sensitive fusion protein ß	Napb	0.0021	1.78	down	14 months
1435396	syntaxin binding protein 6	Stxbp6	0.0290	1.46	down	5 months
1435396	syntaxin binding protein 6	Stxbp6	0.0179	1.33	down	14 months
1429314	synaptotagmin XI	Syt11	0.0274	1.47	down	5 months
1415756	SNAP-associated protein	Snapap	0.0058	1.70	down	5 months
1455304	unc-13 homolog C	Unc13c	0.0070	2.86	down	14 months
1436991	gelsolin	Gsn	0.0092	1.62	down	5 months
1419392	piccolo	Pclo	0.0267	1.47	down	14 months
1451484	synapsin I	Syn1	0.0098	1.49	down	5 months
1435511	synapsin II	Syn2	0.0146	1.74	down	5 months
1435667	regulating synaptic membrane exocytosis 1	Rims1	0.0370	1.32	up	3 months
1422880	synaptophysin-like protein	Sypl	0.0384	1.15	down	3 months
1417919	protein phosphatase 1, regulatory subunit 7	Ppp1r7	0.0256	1.30	down	5 months
1433691	protein phosphatase 1, regulatory subunit 3C	Ppp1r3c	0.0218	1.49	down	5 months
1440285	protein phosphatase 1, regulatory subunit 9A	Ppp1r9a	0.0063	1.24	down	5 months
1452046	protein phosphatase 1, catalytic subunit, gamma	Ppp1cc	0.0276	1.38	down	5 months
1434895	protein phosphatase 1, regulatory subunit 13B	Ppp1r13b	0.0280	1.32	down	14 months
1456072	protein phosphatase 1, regulatory subunit 9A	Ppp1r9a	0.0217	1.32	down	14 months
1440285	protein phosphatase 1, regulatory subunit 9A	Ppp1r9a	0.0304	1.22	down	14 months
1420734	protein phosphatase 1, regulatory subunit 3F	Ppp1r3f	0.0102	1.17	down	14 months
1456606	phosphatase and actin regulator 1	Phactr1	0.0196	1.35	down	5 months
1455101	phosphatase and actin regulator 2	Phactr2	0.0489	1.66	down	5 months
1455101	phosphatase and actin regulator 2	Phactr2	0.0463	1.41	down	14 months

### RNA processing

At late stage disease, GO terms related to gene expression and its regulation are enriched amongst significantly differentially expressed genes, including the sub-categories of mRNA metabolism and processing, and RNA splicing (Table 10). Of the 651 named genes differentially expressed at 14 months, 143 (22%) have functions relating to gene expression, and of these, 132 (92%) are downregulated. This finding is interesting in light of the massive transcriptional downregulation seen at late stage disease.

**Table 10:** Differentially expressed genes in the category 'Gene expression'

Probe ID	Gene Title	Symbol	p value	FC	Regn
1437984	HLA-B-associated transcript 1A	Bat1a	0.0101	1.18	down
1436549	heterogeneous nuclear ribonucleoprotein A1	Hnrpa1	0.0354	1.30	down
1452712	heterogeneous nuclear ribonucleoprotein A3	Hnrpa3	0.0176	1.47	down
1423873	LSM1 homolog, U6 small nuclear RNA associated	Lsm1	0.0450	1.19	down
1434704	myeloid/lymphoid or mixed-lineage leukaemia 5	Mll5	0.0024	1.66	down
1424136	peptidyl prolyl isomerase H	Ppih	0.0123	1.40	down
1451909	PRP4 pre-mRNA processing factor 4 homolog B	Prpf4b	0.0366	1.38	down
1438420	RNA-binding region (RNP1, RRM) containing 2	Rnpc2	0.0004	1.76	down
1459765	splicing factor 1	Sf1	0.0405	1.34	down
1436898	splicing factor proline/glutamine rich	Sfpq	0.0101	1.88	down
1416151	splicing factor, arginine/serine-rich 3	Sfrs3	0.0305	1.24	down
1423130	splicing factor, arginine/serine-rich 5	Sfrs5	0.0494	1.59	down
1416721	splicing factor, arginine/serine-rich 6	Sfrs6	0.0232	1.26	down
1424033	splicing factor, arginine/serine-rich 7	Sfrs7	0.0264	1.30	down
1437180	small nuclear ribonucleoprotein 48	Snrnp48	0.0285	1.68	down
1437007	ubiquitin specific peptidase 39	Usp39	0.0047	1.33	down
1450845	basic leucine zipper and W2 domains 1	Bzw1	0.0269	1.27	up
1437841	cold shock domain containing C2, RNA binding	Csdc2	0.0130	1.43	up
1415920	cleavage stimulation factor, 3' pre-RNA subunit 2, tau	Cstf2t	0.0092	1.27	down
1437071	eukaryotic translation initiation factor 1A, Y-linked	Eif1ay	0.0281	1.21	down
1434538	eukaryotic translation initiation factor 2B, subunit 2 beta	Eif2b2	0.0149	1.23	down
1454664	eukaryotic translation initiation factor 5	Eif5	0.0439	1.12	down
1424252	heterogeneous nuclear ribonucleoprotein D-like	Hnrpdl	0.0046	2.20	down
1415911	imprinted and ancient	Impact	0.0009	1.47	down
1451125	poly(A) binding protein interacting protein 2B	Paip2b	0.0327	1.14	down
1424216	poly (A) polymerase alpha	Papola	0.0286	1.55	down
1427544	poly (A) polymerase alpha	Papola	0.0305	1.22	down
1436586	ribosomal protein S14	Rps14	0.0321	1.25	down
1416065	ankyrin repeat domain 10	Ankrd10	0.0494	1.27	down
1435307	ankyrin repeat domain 34B	Ankrd34B	0.0154	5.84	down
1452342	amyloid beta (A4) precursor protein-binding, family B, member 2	Apbb2	0.0415	1.21	down
1455647	androgen receptor	Ar	0.0248	1.30	down
1420985	ash1 (absent, small, or homeotic)-like	Ash1l	0.0413	1.23	down
1450072	ash1 (absent, small, or homeotic)-like	Ash1l	0.0416	1.22	down
1449947	AT motif binding factor 1	Atbf1	0.0213	1.49	down
1438992	activating transcription factor 4	Atf4	0.0069	1.35	down
1418271	basic helix-loop-helix domain containing, class B5	Bhlhb5	0.0456	1.69	down
1452850	breast cancer metastasis-suppressor 1-like	Brms1l	0.0371	1.28	down
1435445	cyclin T2	Ccnt2	0.0159	1.52	down
1420497	CCAAT/enhancer binding protein zeta	Cebpz	0.0435	1.28	down
1454641	CGG triplet repeat binding protein 1	Cggbp1	0.0063	1.42	down
1434002	checkpoint supressor 1	Ches1	0.0452	1.37	down
1438255	checkpoint supressor 1	Ches1	0.0459	1.41	down
1436980	CCR4-NOT transcription complex, subunit 2	Cnot2	0.0089	1.36	down
1456576	CCR4-NOT transcription complex, subunit 2	Cnot2	0.0046	1.31	down
1437982	COX15 homolog, cytochrome c oxidase assembly protein	Cox15	0.0406	1.14	up
1452901	cAMP responsive element binding protein 1	Creb1	0.0148	1.44	down
1436983	CREB binding protein	Crebbp	0.0428	1.23	down
1452857	CREB/ATF bZIP transcription factor	Crebzf	0.0245	1.45	down
1454931	CREBBP/EP300 inhibitory protein 2	Cri2	0.0384	1.24	down
1429618	cylindromatosis (turban tumor syndrome)	Cyld	0.0025	1.55	down
1448234	DnaJ (Hsp40) homolog, subfamily B, member 6	Dnajb6	0.0214	1.23	down
1459805	dihydrouridine synthase 3-like	Dus3l	0.0103	1.39	down
1418850	enhancer of polycomb homolog 1	Epc1	0.0133	1.23	down
1455267	estrogen-related receptor gamma	Esrrg	0.0026	1.50	down
1456615	fetal Alzheimer antigen	Falz	0.0130	1.33	down
1423709	phenylalanine-tRNA synthetase-like, beta subunit	Farslb	0.0270	1.34	down
1459861	F-box and leucine-rich repeat protein 10	Fbxl10	0.0052	1.32	down
1428890	fem-1 homolog c	Fem1c	0.0498	1.22	down
1417113	germ cell-less homolog	Gcl	0.0341	1.31	down
1424296	glutamate-cysteine ligase, catalytic subunit	Gclc	0.0143	1.38	down
1448381	G elongation factor 1	Gfm1	0.0045	1.35	down
1437163	general transcription factor II H, polypeptide 4	Gtf2h4	0.0446	1.17	up
1425628	transcription factor TFII-I-alpha	Gtf2i	0.0296	1.32	down
1416176	high mobility group box 1	Hmgb1	0.0077	1.29	down
1455626	homeo box A9	Hoxa9	0.0190	1.61	down
1454760	HIV TAT specific factor 1	Htatsf1	0.0337	1.28	down
1460669	interleukin enhancer binding factor 3	Ilf3	0.0213	1.27	down
1455762	kinase D-interacting substrate 220	Kidins220	0.0152	1.26	down
1456341	Kruppel-like factor 9	Klf9	0.0080	1.38	down
1455214	microphthalmia-associated transcription factor	Mitf	0.0081	1.30	down
1435547	MKL/myocardin-like 2	Mkl2	0.0335	1.35	down
1443500	myeloid/lymphoid or mixed lineage-leukemia translocation to 10	Mllt10	0.0080	1.16	up
1457632	myeloid ecotropic viral integration site-related gene 1	Mrg1	0.0333	2.40	down
1424204	mitochondrial ribosomal protein L13	Mrpl13	0.0101	1.24	down
1434971	mitochondrial ribosomal protein L15	Mrpl15	0.0463	1.13	down
1440989	mitochondrial ribosomal protein L35	Mrpl15	0.0041	1.35	down
1456109	mitochondrial ribosomal protein S15	Mrps15	0.0311	1.30	down
1452608	c-myc binding protein	Mycbp	0.0004	1.47	down
1423201	nuclear receptor co-repressor 1	Ncor1	0.0107	1.26	down
1447693	Neogenin	Neo1	0.0410	1.18	down
1448963	nuclear transcription factor-Y gamma	Nfyc	0.0215	1.19	down
1434398	NF-kappaB repressing factor	Nkrf	0.0074	1.71	down
1419112	nemo like kinase	Nlk	0.0478	1.27	down
1416958	nuclear receptor subfamily 1, group D, member 2	Nr1d2	0.0079	1.57	down
1454851	nuclear receptor subfamily 2, group C, member 2	Nr2c2	0.0491	1.15	down
1448493	polyadenylate-binding protein-interacting protein 2	Paip2	0.0261	1.42	down
1426878	polybromo1	Pbrm1	0.0033	1.22	down
1427266	polybromo1	Pbrm1	0.0046	1.31	down
1417493	polycomb group ring finger 4	Pcgf4	0.0091	1.24	down
1453271	PHD finger protein 14	Phf14	0.0151	1.52	down
1456395	peroxisome proliferative activated receptor, γ, coactivator 1a	Ppargc1a	0.0001	1.66	down
1456037	prolactin regulatory element binding	Preb	0.0147	1.41	down
1428254	purine rich element binding protein B	Purb	0.0102	1.29	down
1436979	RNA binding motif protein 14	Rbm14	0.0106	1.19	down
1422660	RNA binding motif protein 3	Rbm3	0.0398	1.54	up
1443922	REST corepressor 3 (Rcor3), mRNA	Rcor3	0.0495	1.32	up
1434521	regulatory factor X domain containing 2 homolog	Rfxdc2	0.0103	1.39	down
1438505	ribonuclease III, nuclear	Rnasen	0.0341	1.14	down
1426660	ribosomal protein L23a	Rpl23a	0.0332	1.26	down
1437975	ribosomal protein L23a	Rpl23a	0.0488	1.18	down
1436046	ribosomal protein L29	Rpl29	0.0144	1.15	down
1448846	ribosomal protein L29	Rpl29	0.0314	1.20	down
1454627	ribosomal protein L29	Rpl29	0.0113	1.38	down
1455348	ribosomal protein L29	Rpl29	0.0203	1.21	down
1420381	ribosomal protein L31	Rpl31	0.0134	1.40	down
1438986	ribosomal protein S17	Rps17	0.0496	1.24	down
1430288	ribosomal protein S21	Rps21	0.0471	1.19	down
1415876	ribosomal protein S26	Rps26	0.0311	1.33	down
1423763	ribosomal protein S28	Rps28	0.0406	1.15	down
1435816	ribosomal protein S6	Rps6	0.0011	1.15	up
1448584	arginine/serine-rich coiled-coil 1	Rsrc1	0.0148	1.25	down
1428219	RING1 and YY1 binding protein	Rybp	0.0223	1.46	down
1416008	special AT-rich sequence binding protein 1	Satb1	0.0242	1.28	down
1417892	sirtuin 3 (silent mating type information regulation 2, homolog) 3	Sirt3	0.0200	1.27	down
1426668	solute carrier family 30 (zinc transporter), member 9	Slc30a9	0.0325	1.34	down
1429624	SAFB-like transcription modulator	Sltm	0.0193	1.21	down
1436703	small nuclear RNA activating complex, polypeptide 2	Snapc2	0.0432	1.14	up
1444531	Superoxide dismutase 2, mitochondrial	Sod2	0.0254	1.21	up
1451542	single-stranded DNA binding protein 2	Ssbp2	0.0262	1.40	down
1434238	RNA polymerase II, TATA box binding protein-associated factor	Taf2	0.0063	1.54	down
1436318	TAR DNA binding protein	Tardbp	0.0104	1.60	down
1452593	transcription elongation factor B (SIII), polypeptide 1	Tceb1	0.0360	1.15	up
1421147	telomeric repeat binding factor 2	Terf2	0.0355	1.24	down
1460545	thyroid hormone receptor associated protein 3	Thrap3	0.0201	1.11	down
1416812	cytotoxic granule-associated RNA binding protein 1	Tia1	0.0287	1.47	down
1416814	cytotoxic granule-associated RNA binding protein 1	Tia1	0.0115	1.50	down
1434898	trinucleotide repeat containing 6a	Tnrc6a	0.0004	1.57	down
1434899	trinucleotide repeat containing 6a	Tnrc6a	0.0039	1.43	down
1438376	tripartite motif protein 27	Trim27	0.0415	1.15	down
1426954	tripartite motif protein 33	Trim33	0.0320	1.27	down
1447780	Tu translation elongation factor, mitochondrial	Tufm	0.0433	1.16	down
1435389	ubiquitin A-52 residue ribosomal protein fusion product 1	Uba52	0.0105	1.35	down
1455222	upstream binding protein 1	Ubp1	0.0403	1.35	down
1427097	WW domain containing E3 ubiquitin protein ligase 1	Wwp1	0.0464	1.25	down
1420011	X-box binding protein 1	Xbp1	0.0167	1.24	down
1437223	X-box binding protein 1	Xbp1	0.0496	1.33	down
1422569	YY1 transcription factor	Yy1	0.0313	1.35	down
1457285	zinc finger protein 187	Zfp187	0.0332	1.20	down
1426895	zinc finger protein 191	Zfp191	0.0081	1.18	down
1456824	zinc finger protein 612	Zfp612	0.0388	1.16	down
1437873	zinc finger protein 799	Zfp799	0.0078	1.48	down
1448875	zinc fingers and homeoboxes protein 1	Zhx1	0.0471	1.18	down
1426531	zinc finger, MYND domain containing 11	Zmynd11	0.0005	1.32	down

Among the changes seen in this functionally category of genes, was downregulation of a number of splicing factors, and other genes involved in mRNA processing. Several ribosomal components also showed reduced expression. The cAMP responsive element binding protein, *CREB1 *is a stimulus-inducible transcription factor which mediates nuclear responses underlying the development, function and plasticity of the nervous system [80]. Downregulation of several members of the CREB family is seen in VEGF^δ/δ ^mice-*CREB1*, *CREBBP*, *CREBzf*, its interacting partner, *ATF4 *(*CREB2*), and *Cri2*. Inactivation of neuronal CREB1 has been shown to cause defects in neurone migration, similar to those observed with reelin [81], and to stimulate neurodegeneration [82]. There is also downregulation of the androgen receptor, and related nuclear receptors, *Nr1d2*, *Nr2c2 *and the oestrogen receptor, *Esrrg*. Steroid hormones have wide ranging effects on the structure and function of the nervous system. Androgen receptor function is known to be important for motor neurone survival, and disruption of this function in Kennedy's disease contributes to the motor neurone degeneration seen in this condition [83].

### Verification of microarray results by QPCR

The results of QPCR verification for the 15 representative genes chosen is shown in Table 11. Eight genes showed a significant downregulation of expression in VEGF^δ/δ ^mice, which supported the microarray findings. A further 4 showed a trend towards downregulation which failed to reach significance, while 4 showed no change in expression despite a finding of significant expression by microarray analysis. The majority of microarray publications have indicated that arrays and QPCR analysis usually support each other qualitatively. However, it is well recognized that significant quantitative differences occur between microarray and QPCR data [84]. This may be related to gene specific variation in the hybridization kinetics associated with the two technologies, low fold changes or hybridization signals in the microarray experiment, or lack of transcript concordance between the probes used for microarray and QPCR analysis. The proportion of genes validated by this study using QPCR, with the larger sample sizes at 3 and 5 months, is comparable to that found by other studies [85,86]. Although not all changes seen on microarray were validated by QPCR, it has been argued that where the focus of microarray analysis is the overall pattern of gene expression rather than the response of a few genes, as in this study, there is less utility in confirming the expression differences of individual genes [87].

**Table 11:** Comparison of QPCR and microarray data of the 15 genes selected for verification

		Microarray result		QPCR result		
**Time point**	**Gene Symbol**	**FC**	**P value**	**n**	**FC**	**P value**

**3 months**	Zfp101	+4.35	0.01	6	No change	
	**Fos**	-2.32	0.03	6	**-2.59**	**0.0003**
	**Reelin**	-1.88	0.004	6	**-1.68**	**0.03**
	HspA5	-1.44 to -1.46	0.02 to 0.01	6	-1.34	0.06

**5 months**	**Fos**	-2.17	0.01	6	**-2.13**	**0.0007**
	**Ldlr**	-2.14	0.02	6	**-1.42**	**0.006**
	**Scd1**	-2.07	0.02	6	**-1.19**	**0.01**
	Nrp1	-1.58 to -2.02	0.02 to 0.009	6	No change	
	Mtap1B	-2.10	0.01	6	No change	
	**Alcam**	-1.60 to -1.94	0.03 to 0.02	6	**-1.43**	**0.01**

**14 months**	**Reelin**	-2.41	0.007	3	**-2.1**	**0.008**
	HSPA5	-1.48	0.004	3	-1.27	0.2
	Hnrpdl	-2.20	0.005	3	-1.21	0.2
	Tnrc6a	-1.43 to -1.57	0.004 to 0.0004	3	No change	
	**Alcam**	-1.50 to -1.65	0.04 to 0.004	3	**-1.59**	**0.003**

Significant results are highlighted in bold; n = no. of transgenic mice used for QPCR verification, with an equal number of gender matched littermate controls.

### Reduced axon growth of VEGF^δ/δ ^motor neurones grown in vitro

**Figure 6:**
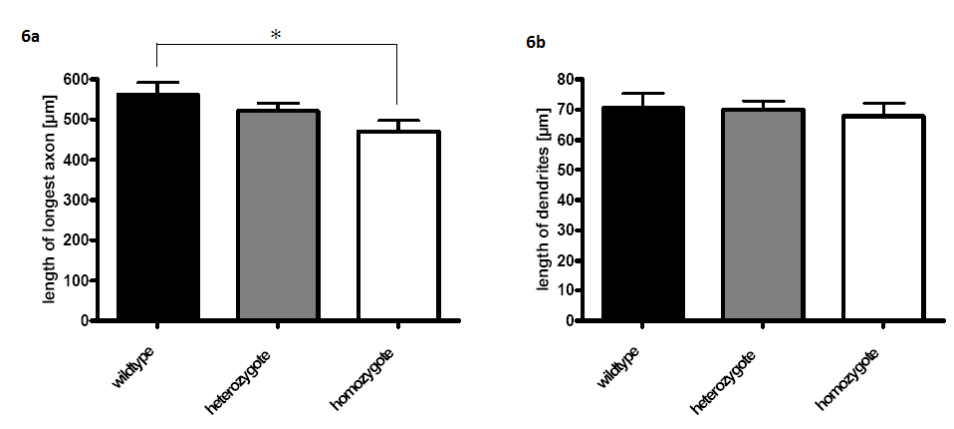
**The average length of the longest axonal process observed in primary motor neuron cultures isolated from E12.5VEGF^δ/δ ^(n = 4), VEGF^δ/wt ^(n = 14) and VEGF^wt/wt ^(n = 8) mice (Figure 6a)**. These motor neurons were grown for 7 days on laminin with BDNF and CNTF. The average length of dendrites isolated from the same mice is also shown (Figure 6b). Asterisk indicates p < 0.05.

Downregulation of genes involved in nervous system development and axonogenesis was observed in motor neurones of VEGF^δ/δ ^mice. A functional correlation of this finding was demonstrated by the growth of primary motor neurones from VEGF^wt/wt^, VEGF^δ/*wt *^and VEGF^δ/δ ^mice *in vitro*. Survival of primary motor neurone cultures grown under basal conditions for 7 days was unaffected by deletion of the hypoxia response element of the VEGF gene (data not shown). A significant reduction in the length of axons was observed in cultures of motor neurones homozygous for the HRE deletion, compared to wild-type motor neurones (472 ± 26 μm vs 562 ± 31 μm, p = 0.047; figure 6a). The outgrowth of dendrites was unaffected (p = 0.34; figure 6b).

## Discussion

### Upregulation of oxidative phosphorylation in presymptomatic mice

A small but significant upregulation of genes involved in the TCA cycle and oxidative phosphorylation was observed in VEGF^δ/δ ^mice in presymptomatic stages. This finding is not in keeping with the 'chronic ischaemia' hypothesis of neurodegeneration in the VEGF^δ/δ ^mouse model: were motor neurones in a state of chronic oxygen deprivation due to reduced baseline neural blood flow, oxidative phosphorylation would have been downregulated. A similar upregulation of oxidative phosphorylation was seen in motor neurones in the SOD1 mouse model of ALS [21], and this may be a non-specific adaptation to cellular stress in the early stages of disease.

### Transcriptional downregulation and changes in the regulation of gene expression

One of the most marked changes observed in the VEGF^δ/δ ^mouse model was transcriptional downregulation with disease progression, which was accompanied in late stage disease by a reduction in expression of genes involved in the regulation of gene expression, particularly mRNA processing. Similar transcriptional downregulation was observed in a study of the SOD1 mouse model of ALS [21], and in the ageing human brain [88]. The cause of this transcriptional downregulation is not known, although several mechanisms may be proposed. Firstly, it may be due to low level oxidative modification of nuclear DNA, which has been shown to accompany downregulation of gene expression in the ageing human brain [88]. Secondly, reduction in expression of VEGF could result in reduced induction of sequence-specific transcription factors, such as Fos, which was robustly downregulated in this study, with consequent downregulation of genes regulated by those factors [89,90]. Thirdly, epigenetic modifications which cause suppression of gene expression, such as DNA methylation, increase with age and are accelerated in neurodegenerative diseases [91].

### Downregulation of cholesterol metabolism at disease onset

Five genes in the final committed pathway to cholesterol synthesis, and two receptors which bind cholesterol and transport it into the cells, were downregulated in the VEGF^δ/δ ^mouse. Neuronal cholesterol is either synthesised by neurones or produced by astrocytes, bound to apolipoprotein E (APOE), and taken up by neurones via the low density lipoprotein receptor[92]. Cholesterol-rich lipid rafts in the growth cone are required for downstream signaling of adhesion molecules and guidance receptors during axon growth and guidance. Cholesterol stimulates the formation of synapses, and has important roles in synaptic function, and the release of neurotransmitters [93-96]. A recent study showed that the neurotrophic factor, BDNF, promotes synaptic development in cortical neurones via the stimulation of cholesterol biosynthesis [97], and the findings of this study suggest that VEGF may have a similar function. Cholesterol homeostasis is already implicated in the pathogenesis of neurodegenerative disease. There is a strong association between Alzheimer's disease and the APOE4 allele, which has reduced efficacy in cholesterol delivery to cells, and in stimulating neurite outgrowth [98]. Downregulation in the expression of genes involved in the cholesterol synthesis pathway is seen in cortical tissue of patients with Huntington's disease, and in mouse and cell models of the disease [99,100].

### Neurite outgrowth and synaptogenesis

In adult mice, reduction in neural expression of VEGF, through deletion of its hypoxia-response element, causes downregulation of genes which are known to play a role in the growth of neuronal processes and formation of synapses during embryonic development. These include promoters of outgrowth of neuronal processes, components of neurotrophic signalling pathways, cell adhesion molecules, and the cholesterol synthesis pathway. The most significantly enriched functional gene categories at 5 months relate to axon extension and axon maintenance. This finding was supported by *in vitro *data: primary motor neurones from VEGF^δ/δ ^mice show a significant reduction in axon outgrowth compared to their wild-type littermates. This effect of VEGF was specific to that neuronal compartment, with no effect on dendrite growth, or on cell survival. Exogenous VEGF protein stimulates axon outgrowth in cultures of primary motor neurones [20] although to a lesser degree than exogenous BDNF and CNTF. Despite addition of BDNF and CNTF to the primary neurone cultures in this study, axon outgrowth from VEGF^δ/δ ^motor neurones was significantly reduced, indicating that the effect of VEGF on axonal outgrowth cannot be substituted for by the presence of other growth factors.

Differential expression of genes relating to the growth of neuronal processes, and formation of synapses was an unexpected finding in the adult animal, but there is growing evidence that changes involved in the plasticity of the nervous system and in the maintenance of neuronal networks in the adult animal recapitulate, to some degree, those underlying the formation of neuronal networks during development: cell adhesion molecules that mediate target recognition and synapse formation during embryogenesis also lead to changes in synaptic efficacy in the adult nervous system [101]; BMP signalling at the Drosophila NMJ is not only required for normal synaptic growth, but also for synaptic stabilization, via LIM kinase-1, and disruption of this pathway leads to synaptic disassembly and retraction [102]; the wnt signalling pathway maintains activity-dependent axon stability in adult olfactory neurones [103]; neurotrophins were first recognized as target-dependent survival factors for developing neurones during embryogenesis, but they have also been shown to promote synaptic stability and maintain neuronal processes in response to mechanical axonal injury [104].

Although the NMJ is considered a relatively stable structure, plasticity of the motor system is seen in response to changing physiological demands and to pathological conditions. Alterations in synaptic structure and function occur in the motor cortex, spinal cord and NMJ in response to exercise [105-107], while partial denervation or paralysis results in sprouting and reinnervation from adjacent nerve fibres [57]. The observation of fibre-type grouping in ageing muscle indicates that denervation and reinnervation of muscle fibres occurs with normal ageing [108]. Motor units differ in the plasticity of their synapses. Synapses formed on Fast Synapsing (FaSyn) muscle, or by fast-fatiguable (FF)-type motor units exhibit relatively little synaptic plasticity, compared to Delayed Synapsing (DeSyn) or slow (S)-type motor units. Motor units with less synaptic plasticity exhibit early susceptibility to loss in motor neurone degenerative disease, and in the SOD1 mouse model of ALS, a progressive impairment of stimulus-induced synaptic sprouting was observed over the course of the disease, suggesting that the absence of synaptic plasticity, and disease-induced synaptic loss are mechanistically linked [109,110].

The transcriptional changes observed in VEGF^δ/δ ^mice would be predicted to result in a reduced capacity for the morphological adaptations that occur during plasticity of the motor unit, resulting in increased vulnerability to degeneration. As neuronal processes and synapses are required continuously to retract and reform during the process of synaptic plasticity, downregulation of genes involved in neurite growth and synapse formation would be likely to result in gradual attrition of synapses and distal cellular processes. Synapse retraction causes loss of access of the neurone to trophic signals from target tissue. A recent study has shown that muscle hypermetabolism is sufficient to cause degeneration of NMJs, and subsequent loss of spinal cord motor neurones [111]. Although ALS is characterized pathologically by loss of motor neurone somata, and gliosis in the anterior horn of the spinal cord, these post mortem findings may not reflect changes occurring earlier in the disease. There is evidence in both human ALS and in mouse models, that motor neurone death occurs by the type of 'dying back' axonal degenerative pathophysiology, that would be predicted in this model, with defects of neuromuscular transmission, end-plate denervation, and 'peripheral pruning' of axons as the earliest observed events [112-114]. VEGF^δ/δ ^mice similarly show relative preservation of spinal motor neurones, at stages of disease where there is marked axonal loss [1].

The relevance of this finding in the VEGF^δ/δ ^mouse model of ALS to the human disease is unknown, but failure of synaptic plasticity has been proposed as a pathogenic mechanism in Alzheimer's disease [115]. A failure to maintain neuronal processes and synapses in the face of increasing demands of neuronal plasticity would explain two epidemiological observations in ALS: the associations between exposure to vigorous physical activity [116,117] or skeletal fractures [118], and the risk of developing the disease.

An alternative hypothesis is that the pathogenic insult in VEGF^δ/δ ^mice occurs during their development, when a lack of VEGF may lead to aberrant neuronal guidance and formation of neural networks. Subtle defects in neuronal positioning and in synaptic circuitry in VEGF^δ/δ ^mice could result in reduced stability of synaptic connections, or a reduction in target-derived neurotrophic support, with consequent increased loss of motor neurones during ageing. This hypothesis is supported by the observation that embryonic motor neurones show a reduction in axonal outgrowth *in vitro*.

## Conclusions

Reduction in expression of VEGF, through deletion of a regulatory promoter region of the gene, results in adult-onset motor neurone degeneration that resembles human ALS. We have presented evidence here that this phenotype is accompanied by reduction in expression, from symptom onset, of the cholesterol synthesis pathway, and genes involved in nervous system development, including axonogenesis, synapse formation, growth factor signalling pathways, cell adhesion and microtubule-based processes. These findings raise the possibility that VEGF is required for the maintenance of distal neuronal processes in the adult animal, perhaps through promotion of remodelling of distal processes and synapses in the face of the demands of neuronal plasticity. A reduction in VEGF expression in VEGF^δ/δ ^mice may lead to failure of the maintenance of neuronal circuitry, causing axonal retraction and cell death.

## Authors' contributions

AB carried out the laser capture microdissection, RNA processing and microarray analysis and QPCR studies, and drafted the manuscript; PH participated in the study design and in the microarray analysis; HH participated in the laser capture microdissection, microarray analysis and QPCR studies; PK carried out the preparation of neural tissue from VEGF^δ/δ ^mice for laser capture microdissection; FB carried out the *in vitro *studies of axon outgrowth and survival; FC participated in the design of the study, maintenance of the VEGF^δ/δ ^mouse colony, selection and preparation of mice for the study; DL and MS conceived, designed and supervised the *in vitro *studies of axon outgrowth and survival, and DL helped to draft the manuscript; PC and PJS conceived of the study, and participated in its design and coordination, PJS helped to draft the manuscript. All authors read and approved the final manuscript.
